# Antiseizure medication in patients with meningioma: a retrospective cohort study on the long-term impact on depression, anxiety and neurocognitive functioning

**DOI:** 10.1007/s11060-025-05025-w

**Published:** 2025-06-06

**Authors:** L. Laribi, J. C. C. Scheepens, A. H. Zamanipoor Najafabadi, M. J. Vos, W. R. van Furth, S. M. Peerdeman, M. J. B. Taphoorn, P. B. Van der Meer, J. A. F. Koekkoek, the Dutch Meningioma Consortium, L. Laribi, N. R. Biermasz, F. W. Boele, L. Dirven, M. Klein, W. A. Moojen, J. C. Reijneveld, M. J. T. Verstegen

**Affiliations:** 1 Department of Neurology, Leiden University Medical Center, Postbus 9600, Leiden, 2300 RC the Netherlands; 2 Department of Ophthalmology, Leiden University Medical Center, Leiden, the Netherlands; 3 Department of Neurology, Haaglanden Medical Center, The Hague, the Netherlands; 4 Department of Neurosurgery, Leiden University Medical Center, Leiden, the Netherlands; 5 Department of Neurosurgery, Amsterdam University Medical Center-VU University, Amsterdam, the Netherlands; 6 Department of Psychiatry, University Medical Center Utrecht, Utrecht, The Netherlands

**Keywords:** Antiseizure medication (ASM), Anxiety, Brain Tumor-Related Epilepsy (BTRE), Depression, Meningioma, Neurocognitive functioning

## Abstract

**Background:**

Patients with meningiomas often suffer from brain tumor-related epilepsy for which they are prescribed antiseizure medication (ASM). ASMs have been associated with neuropsychiatric side effects such as depression, anxiety, and cognitive impairments. However, the association between ASM use and mood and cognition in meningioma patients remains unclear. In this study, we aimed to investigate the association of ASM use, and specifically the use of levetiracetam, with depression, anxiety, and neurocognitive functioning.

**Methods:**

In this multicentre retrospective study, data from 187 meningioma patients were collected from neurocognitive tests, the HADS questionnaire, and medical records. Multivariable logistic regression analyses were used to evaluate the association between ASM use, and depression, anxiety and neurocognitive impairment. Potential confounders were included based on the existing literature. Due to sample size limitations, the association of levetiracetam use with depression, anxiety and neurocognitive impairment could not be statistically analyzed.

**Results:**

The prevalence of depression, anxiety and cognitive impairment did not differ significantly for patients using ASM (*n* = 41) as compared to patients not using ASM (*n* = 146) (aOR = 0.81; 95% CI 0.26–2.54; aOR = 0.63; 95% CI 0.22–1.82; aOR = 1.42; 95% CI 0.51–3.98).

**Conclusion:**

Our findings show no significant association between ASM use and mood and neurocognitive dysfunction in meningioma patients.

**Supplementary Information:**

The online version contains supplementary material available at 10.1007/s11060-025-05025-w.

## Introduction

Meningioma represents approximately 20–30% of primary intracranial tumors diagnosed in 2023, making it the most common intracranial tumors in adults [1, 2]. Meningiomas arise from cells making up the meninges, which comprise connective tissue sheaths directly around the brain, thus providing support to the brain [3]. These tumors, which are usually low-grade, with over 95% of meningioma patients having a non-malignant World Health Organization (WHO) grade 1 or 2 tumor [4], may become symptomatic depending on the location and size of the tumor. These symptoms include seizures as well as changes in mood, behavior, and cognition [5–7].

In total, more than one third of patients with meningioma experience epileptic seizures over the course of the disease [8]. Epilepsy is associated with cognitive deficits and psychiatric symptoms such as depression and anxiety [9]. In patients with intracranial tumors, guidelines recommend prescribing antiseizure medication (ASM) after a single seizure [10]. Levetiracetam is the most commonly used ASM in brain tumor-related epilepsy as it does not interact with other drugs, such as chemotherapy or other agents inducing the cytochrome P450 system [11, 12]. This is especially important in glioma patients, who receive chemotherapy more commonly than meningioma patients [13]. Therefore, theoretically, enzyme-inducing ASMs present a lower risk of drug interaction for meningioma patients than for glioma patients [14].

Conflicting evidence exists on the correlation between ASM use and neuropsychiatric and neurocognitive adverse effects in patients with intracranial tumors, with limited available research on meningiomas. Whereas levetiracetam has often been linked to anxiety and depression in patients with epilepsy, valproic acid and lamotrigine could be effective as mood-stabilizers. Levetiracetam seems to improve cognition, whereas enzyme-inducing ASMs like phenytoin are associated with cognitive dysfunction [15–18]. On the contrary, one study conducted in patients with glioma and epilepsy, described no association between ASM use, particularly levetiracetam, and the development of depression, anxiety, or subjective cognitive impairment [19].

Determining the impact of ASM on mood and cognition in meningioma patients is important to make well informed decisions on which ASM to use. To this day, sparse evidence exists regarding the impact of ASM use on mood and cognition in meningioma patients, although meningioma patients widely use ASM [11]. In fact, previous research on the effect of mood and cognition has mostly focused on patients with gliomas or primary intracranial tumors in general, and rarely solely on patients with meningioma [19, 20]. Since the standards of antitumor treatment differ per type of brain tumor, with chemotherapy being more frequently used in glioma, previous studies on adverse effects of ASM are poorly generalizable to the meningioma population [12, 13].

In this study, we aimed to evaluate the influence of ASM use, and specifically the use of levetiracetam, on depression, anxiety and neurocognitive functioning in patients with meningioma. Based on limited available evidence, we hypothesized that the use of ASM was independently associated with self-reported depression, anxiety, and cognitive impairment in meningioma patients. Furthermore, we hypothesized that the use of levetiracetam was independently associated with self-reported depression, anxiety, and improvement of neurocognitive functioning in meningioma patients compared to the use of other commonly prescribed ASMs.

## Methods

We performed a cross-sectional retrospective analysis of data collected from Leiden University Medical Center (LUMC), Haaglanden Medical Center (HMC) and Amsterdam University Medical Center (AUMC) using different tests and questionnaires to analyze neurocognitive functioning, depression and anxiety in patients with meningioma. All data from questionnaires and patients’ medical history were gathered within a database. Data collection was ethically approved in all participating centers. The methods for this study were described previously in more detail [21]. This study was conducted according to the STROBE guidelines (Supplementary Table 1).

### Population

The study included 187 patients (≥ 18 years) with a histologically confirmed or suspected intracranial meningioma, who completed primary antitumor treatment at least 5 years prior to the beginning of the data collection. Patients had consented to complete a neurocognitive test battery to assess neurocognitive functioning and questionnaires to assess self-reported depression and anxiety from July, 2016 to April, 2019. As antitumor treatment, the patients received surgical resection and/or radiotherapy. Additional information was retrieved concerning their age, sex, tumor characteristics (i.e., WHO grade, location, and size), Karnofsky Performance Status (KPS), date of diagnosis, number of seizures in the last three months, supportive treatment, comorbidities, and complications after treatment. Use of ASM and other prescription medications at baseline was also retrieved from the medical records. Patients were excluded from the study if they (1) received whole-brain radiotherapy for a disease other than a confirmed or suspected intracranial meningioma, (2) were diagnosed with neurofibromatosis type 2, as this may substantially influence the disease trajectory of meningiomas, (3) were diagnosed with a neurodegenerative disease influencing their cognitive abilities, or (4) had insufficient understanding of the Dutch language.

### Procedures

For data collection regarding neurocognitive functioning, depression, and anxiety, meningioma patients were asked to complete several questionnaires and to undergo a neuropsychological assessment once.

#### Neurocognitive functioning

Neurocognitive functioning was assessed with a group of tests covering six neurocognitive domains that may be affected in intracranial tumor patients (Table 1). Z-scores were calculated for each of these domains using a reference sample from the Maastricht Aging Study. A cutoff of ≤ -1.5 for these Z scores on each of these domains was regarded as a neurocognitive deficit in that domain. A neurocognitive deficit in at least two of these domains was considered clinically relevant cognitive impairment.

**Table 1:** Tests used to assess neurocognitive function and their respective neurocognitive domains

Verbal Memory	The Rey Auditory Verbal Learning Test (AVLT) [39]
Executive functioning and psychomotor functioning	The Concept Shifting Test (CST) [40] Categoric Word Fluency Test (CWFT) [41]
Working memory	The Sternberg’s Memory Scanning Test (MST) [42]
Processing speed	The Concept Shifting Test (CST) [43] The Digit-Symbol Substitution Test (DSST) [42, 43]
Information processing	The Digit-Symbol Substitution Test (DSST) [42, 43]
Attention	The Stroop Colour-Word Test (SCWT) [44]

#### Depression and anxiety

The Hospital Anxiety and Depression Scale (HADS) was used to determine the levels of anxiety and depression in participants [22]. Patients who had a score of ≥ 8 points on the depression or anxiety subscale were classified as depressed or anxious, respectively.

#### Medication use

We collected information on patients’ use of prescription medications, excluding ASMs, with > 1% risk of developing depression, anxiety, and/or cognitive (i.e., neuropsychiatric and/or neurocognitive) adverse effects based on a Dutch evidence-based online tool which provides comprehensive information on drug efficacy, safety, indications, and prescribing guidelines, *Farmacotherapeutisch Kompas* [23]. Patients were classified as using either none or at least one medication, for the three adverse effects combined (Supplementary Table 2).

### Statistical analysis

First, a non-response analysis using the chi-square test was conducted on baseline characteristics of patients. 450 eligible patients who refused to participate in the study were included in the non-responder group. Following this, a T-test was used to compare point prevalence rates of depression and anxiety between meningioma patients and normative data [22].

The Directed Acyclic Graph (DAG) representation was used to establish potential confounders based on the literature. To assess the association between use of ASM and depression, anxiety and neurocognitive impairment, univariable logistic regression analyses were performed. In addition, univariable logistic regression analyses were performed with potential confounders. Furthermore, multivariable logistic regression analyses were done to assess the association between ASM and depression, anxiety and cognitive impairment after correction for confounders. Because this study is an aetiological study, the confounders were entered in the multivariable logistic regression based on clinical relevance determined using literature and expert opinions regardless of their statistical significance in the univariable analysis [24]. According to the Rule of Ten Events per Variable (EPV), a maximum of six confounders was included in the analysis due to the sample size limitations in order to avoid bias and instability in the regression coefficients [25]. The selected confounders (next to ASM use) were age, WHO grade, treatment, number of seizures in the last three months, and use of medication with neuropsychiatric and/or neurocognitive adverse effects. If one of the potential confounders could not be included in a multivariable analysis due to lack of variability in the sample (i.e., all patients had the same value for that variable), the odds ratio for that confounder was listed as “N/A” (not applicable) in the results table.

Multicollinearity was assessed by calculating the variance inflation factor (VIF) and variance decomposition proportion (VDF), with cutoffs set to > 5 and > 0.8 respectively [26]. Finally, we performed sensitivity analyses with different cutoffs for anxiety, depression and cognitive impairment. These cutoffs were ≥ 5 and ≥ 10 for depression and anxiety and deficits in ≥ 1 and ≥ 3 neurocognitive domains for cognitive impairment.

Rates of depression, anxiety, and cognitive impairment were described in patients using levetiracetam versus those using other or no ASMs. However, the sample size of the levetiracetam group was too small to allow for formal statistical analysis.

We used SPSS 29.0 for Windows for all statistical analyses, and a p-value < 0.05 was considered statistically significant.

## Results

A total of 187 participants were included in the study, of which 41 individuals were using ASM (22%) at the time of the study and 146 individuals were not (78%). Of the 41 patients using ASM, 14 used levetiracetam (34%), 11 used carbamazepine (27%), 9 used valproic acid (22%), 8 used lamotrigine (20%). In total, 10 patients received polytherapy (24%). The majority of participants were female (79%), had a level of education below university level (66%), and had comorbidities (60%). Furthermore, most patients had a WHO grade 1 tumor (78%) and underwent surgery instead of radiotherapy (88% vs. 18%). Overall, the vast majority of patients had undergone at least one of both treatments (93%) (Table 2). In use of ASM and anxiety scores, no patients had missing data. Depression scores were missing in 3/187 (2%) of patients, and 4/187 (2%) of patients had missing data in any of the neurocognitive domains. Data were assumed to be missing at random based on observed patterns.

**Table 2:** Summary of population descriptives

		ASM Use (*N* = 41)	No ASM (*N* = 146)	Total (*N* = 187)
Gender [n, %]	Male (ref.)	12 (29%)	28 (19%)	40 (21%)
	Female	29 (71%)	118 (81%)	147 (79%)
Age at study date (years) [mean, SD]		61.47 (11.0)	63.62 (11.1)	
WHO grade [n, %]	Grade I (ref.)	32 (78%)	114 (78%)	146 (78%)
	Grade II - III	4 (10%)	8 (6%)	12 (6%)
	Suspected meningioma	5 (12%)	24 (16%)	29 (16%)
Tumor location [n, %]	Non-skull base meningiomas (ref.)	25 (61%)	75 (51%)	100 (53%)
	Skull base meningiomas	16 (39%)	70 (48%)	86 (46%)
	Unknown	0 (0%)	1 (1%)	1 (1%)
Education level [n, %]	Primary, secondary or tertiary level (ref.)	27 (66%)	97 (66%)	124 (66%)
	Academic/university level	14 (34%)	44 (30%)	58 (21%)
	Unknown	0 (0.00%)	5 (3%)	5 (3%)
Comorbidities [n, %]	No (ref.)	12 (30%)	60 (41%)	52 (28%)
	Yes	28 (68%)	84 (58%)	112 (60%)
	Unknown	1 (2%)	2 (1%)	3 (2%)
KPS [n, %]	≤ 80 (ref.)	5 (12%)	16 (11%)	21 (11%)
	> 80	34 (83%)	127 (87%)	161 (86%)
	Unknown	2 (5%)	3 (2%)	5 (3%)
Radiotherapy [n, %]	No (ref.)	31 (76%)	122 (84%)	153 (82%)
	Yes	10 (24%)	24 (16%)	34 (18%)
Surgery [n, %]	No (ref.)	4 (10%)	19 (13%)	23 (12%)
	Yes	37 (90%)	127 (87%)	164 (88%)
Number of seizures in the last three months [mean, SD]		1.28 (4.4)	0.00 (0.00)	
	Unknown [n, %]	2 (5%)	3 (2%)	5 (3%)

The sociodemographic and clinical characteristics of the *n* = 187 study population are shown

*ref.* = reference category

### Assessment of Non-Response Bias and comparison of point prevalence rates

A total of 450 meningioma patients were included in a non-responder group for a non-response analysis. The mean age of responders was 63.2 years (SD = 11.1), while the mean age of non-responders was significantly higher (67.0 years, SD = 13.5, p-value < 0.001). The WHO grade of the tumors did not differ significantly between responders and non-responders (Supplementary Table 3).

Furthermore, point prevalence rates of anxiety and depression in the study cohort were compared to normative data [22]. No significant differences were found (p-values of 0.22 and 0.08 for anxiety and depression, respectively).

### Association between ASM use and depression, anxiety and neurocognitive impairment

The univariable analysis of each confounder and the determinant variables of depression, anxiety and cognitive impairment in this study can be found in supplementary Tables 4 and 5. There was no significant multicollinearity observed between the included variables. Sensitivity analysis for alternate cutoffs did not give significantly different results.

#### Depression

Of the 187 included patients, 17% were considered depressed, while 82% were not (*n* = 31 vs. *n* = 153). In the group of non-ASM users, 16% were considered depressed versus 17% in the ASM user group (*n* = 24 vs. *n* = 7). The prevalence of depression did not differ significantly between patients not using ASM (reference) and those using ASM both before and after adjustment for potential confounders (uOR = 1.02; 95% CI 0.41–2.57; aOR = 0.81; 95% CI 0.26–2.54) (Table 3). Of the 14 patients using levetiracetam, 4 showed signs of depression (29%), versus 27 of 173 patients using other or no ASMs (16%).

**Table 3:** Odds ratio of ASM use in the multivariable analysis for each outcome

	uOR	95% CI	*P*-value	aOR	95% CI	*P*-value
Depression^1^	1.02	0.41–2.57	0.965	0.81	0.26–2.54	0.720
Anxiety^1^	0.65	0.26–1.59	0.342	0.63	0.22–1.82	0.397
Cognitive Impairment^2^	0.89	0.40–1.97	0.765	1.42	0.51–3.98	0.505

The unadjusted odds ratios (uOR) and adjusted odds ratios (aOR) with corresponding 95% confidence intervals (CI) and p-values for ASM use with regards to each outcome variable. The covariates included in this analysis included age, WHO grade, treatment, number of seizures in the last three months, and use of medication with neuropsychiatric and/or neurocognitive adverse effects. The R^2^ values for depression, anxiety and cognitive impairment were 0.076, 0.130 and 0.204 respectively

^1^ ≥ 8 on the HADS-D ^2^ ≤ -1.5 in at least 2 of the neurocognitive domains

#### Anxiety

Of the 187 included patients, 22% were considered anxious, while 77% were not (*n* = 42 vs. *n* = 144). In the group of non-ASM users, 24% were considered anxious versus 17% in the ASM user group (*n* = 35 vs. *n* = 7). There was no significant difference in the prevalence of anxiety between ASM users and non-users both before and after adjustment for potential confounders (uOR = 0.65; 95% CI 0.26–1.59; aOR = 0.63; 95% CI 0.22–1.82) (Table 3). Of the 14 patients using levetiracetam, 5 showed signs of anxiety (36%) wile 37 of the 173 remaining patients using other or no ASMs (21%).

#### Cognitive impairment

Of the 187 included patients, 26% showed cognitive impairment, while 74% were not (*n* = 49 vs. *n* = 138). In the group of non-ASM users, 27% showed cognitive impairment versus 24% in the ASM user group (*n* = 39 vs. *n* = 10). The use of ASM was not significantly associated with the prevalence of cognitive impairment both before and after adjustment for potential confounders (uOR = 0.89; 95% CI 0.40–1.97; aOR = 1.42; 95% CI 0.51–3.98) (Table 3).

The effect of ASM use versus no ASM use was also analyzed with regards to each of the neurocognitive domains (i.e., executive functioning and psychomotor functioning, verbal memory, working memory, attention, and information processing speed). Here, the use of ASM showed a significant negative impact on verbal memory after adjustment for all confounders (uOR = 2.29; 95% CI 0.89–5.90; aOR = 5.61; 95% CI 1.71–18.38). There was no significant association between the use of ASM and any other neurocognitive domains (Supplementary Table 5). Of the 14 patients using levetiracetam, 5 showed signs of cognitive impairment (29%) versus 44 in the 173 patients using other or no ASMs (25%).

## Discussion

In this study, we evaluated the association between ASM use and depression, anxiety and neurocognitive functioning in patients with meningioma. Based on the sparse available evidence, we hypothesized that ASM use negatively influences mood and cognition of meningioma patients. However, our results did not corroborate this hypothesis. In fact, we found no significant association between ASM use and any of the three outcomes, suggesting that ASM use is not associated with the development of depression, anxiety, and cognitive impairment in meningioma patients. Only on verbal memory, patients using ASM performed worse than patients not using ASM, whereas, for all five other cognitive domains, patients performed similarly in both groups. A previous study found no association between ASM use and neuropsychiatric or neurocognitive impairments [19], which aligns with our findings. This lack of significant association may be explained by other factors which impact the prevalence of these outcomes, thereby overruling the effect of ASM. These factors include the tumor itself, age, education level and comorbidities of the patient, use of antitumor treatments (e.g. surgical resection and/or radiotherapy) and use of medication with depression, anxiety and/or cognition-related adverse effects [3, 15, 27]. For example, in our study, the use of medication with neuropsychiatric and/or neurocognitive adverse effects were significantly associated with anxiety, which may be due to the fact that many anxiolytic medications provide a risk of cognitive adverse effects. Also, the severity of epilepsy, since epilepsy is known to negatively impact cognition and mental health [9, 28], could have a significant effect on the prevalence of neuropsychiatric adverse effects. As ASM may reduce epilepsy and concomitant adverse effects, the effect of the ASM itself on mood and cognition may be only minor in comparison.

Previous studies have reported an association between ASM use and impaired mood and cognition [15, 16, 18]. Part of the differences between our results and previous research may be explained by sociodemographic differences resulting in a varying predisposition for anxiety, depression, and/or cognitive impairment between populations, since many previous studies have been performed in South European and North American populations [16, 27, 29, 30]. Also, varying methods of measuring outcomes between studies could be a relevant factor explaining the observed differences, since our study used objective measures of cognition while many studies used patient-reported (subjective) outcome measures [19, 20]. As objective and patient-reported measures are complementary outcomes, future research should incorporate both objective and patient-reported outcome measures to accurately assess cognitive functioning in meningioma patients [31, 32].

Due to a small sample size, the association between use of levetiracetam and depression, anxiety and cognitive functioning could not be analyzed. Many previous studies found levetiracetam to be associated with neuropsychiatric adverse effects such as depression and anxiety as well as cognitive impairment in patients with different types of intracranial tumors [15, 16, 27, 30, 33]. Levetiracetam differs from other ASMs by binding the SV2A protein, thereby modulating neurotransmitter release, instead of targets such as sodium and calcium channels and GABA receptors, which could explain why levetiracetam may be associated with more neuropsychiatric adverse effects compared to other ASMs [34]. Also, the activity of levetiracetam towards AMPA-gated currents has been hypothesized as one of the drivers for its depressive effects in previous studies [35]. Some studies have found that levetiracetam significantly improved cognitive function [17, 36, 37], which may be partly due to the alleviation of epilepsy-driven cognitive impairment instead of actual cognitive improvement. The mechanism of action of levetiracetam, which differs from other ASMs, may also play a role in generating this improvement [34].

A major strength of our study is the inclusion of a sample of meningioma patients only, which has hardly been studied before in this context. Also, we included the use of medication with depression, anxiety and/or cognition-related adverse effects as potential confounders in the analyses, thereby eliminating a potential source of bias. Furthermore, we performed a non-response analysis to test if meningioma patients who did and did not participate in the study had similar baseline characteristics. Still, some limitations of this study may also be taken into consideration. The sample size of our study is relatively small compared to previous research, and therefore significant effects of the variables of interest could have been missed due to limited statistical power [16, 27, 30, 33]. Because of the sample size constraints, we could only select a limited number of confounders for our analyses, and we could not analyze the association of use of levetiracetam with depression, anxiety, and neurocognitive impairment. Data on some confounders, such as patients’ psychiatric history, were not collected, which may have caused our results to differ from those described in the literature [16, 30].

Also, the use of medication with depression, anxiety and/or cognition-related adverse effects in our study was self-reported, and therefore may be subject to response bias [38]. To continue, our non-response analysis shows significant differences between responders and non-responders, suggesting that our study population may not be representative of the general meningioma patient population. Additionally, our study did not take into consideration the duration of ASM exposure, thus overlooking possible differences in the short-term and long-term effects of the medication. These limitations may affect the solidity of our conclusions.

To conclude, we found that the use of ASM was not significantly associated with depression, anxiety or cognitive impairment in meningioma patients. This alleviates concerns regarding the burden of neuropsychiatric and neurocognitive adverse effects when prescribing ASM to this patient group. Other factors such as tumor characteristics, comorbidities, and use of other medications may be more related to the development of mood and neurocognitive disorders in meningioma patients. Large cohort studies considering all the previously mentioned variables should be conducted to confirm our results.

## Electronic supplementary material

Below is the link to the electronic supplementary material.

Supplementary Material 1

## Data Availability

The datasets generated during and/or analyzed during the current study are available from the corresponding author on reasonable request.
