# Correction to: Antiseizure medication in patients with meningioma: a retrospective cohort study on the long-term impact on depression, anxiety and neurocognitive functioning

**DOI:** 10.1007/s11060-025-05147-1

**Published:** 2025-07-28

**Authors:** L. Laribi, J. C. C. Scheepens, A. H. Zamanipoor Najafabadi, M. J. Vos, W. R. van Furth, S. M. Peerdeman, M. J. B. Taphoorn, P. B. Van der Meer, J. A. F. Koekkoek, The Dutch Meningioma Consortium, L. Laribi, N. R. Biermasz, F. W. Boele, L. Dirven, M. Klein, W. A. Moojen, J. C. Reijneveld, M. J. T. Verstegen

**Affiliations:** 1 Department of Neurology, Leiden University Medical Center, Postbus 9600, 2300 RC Leiden, The Netherlands; 2 Department of Ophthalmology, Leiden University Medical Center, Leiden, The Netherlands; 3 Department of Neurology, Haaglanden Medical Center, The Hague, The Netherlands; 4 Department of Neurosurgery, Leiden University Medical Center, Leiden, The Netherlands; 5 Department of Neurosurgery, Amsterdam University Medical Center-VU University, Amsterdam, The Netherlands; 6 Department of Psychiatry, University Medical Center Utrecht, Utrecht, The Netherlands

**Correction to: Journal of Neuro-Oncology (2025) 174:227–234** 10.1007/s11060-025-05025-w

In this article, the seven last authors were incorrectly listed as individual authors, whereas they should have been indexed as part of the Dutch Meningioma Consortium.

The original publication has been corrected, with the names of the seven contributors listed in a footnote.

