# Supplementary material for: Antiseizure medication in patients with meningioma: a retrospective cohort study on the long-term impact on depression, anxiety and neurocognitive functioning
Source: J Neurooncol. 2025 Jun 6;174(1):227–34. doi: 10.1007/s11060-025-05025-w (PMC12198320; doi:10.1007/s11060-025-05025-w)
Supplement: Supplementary file 1 — Supplementary Material 1 [file 11060_2025_5025_MOESM1_ESM.docx]

Antiseizure medication in patients with meningioma: a retrospective case-study on the long-term impact on depression, anxiety and neurocognitive functioning

L. Laribi^1^*, J.C.C. Scheepens^1^*, A.H. Zamanipoor Najafabadi^2^, M.J. Vos^1,3^, W.R. van Furth^4^, S.M. Peerdeman^5^, M.J.B. Taphoorn^1^, P.B. Van der Meer^1,6^, J.A.F. Koekkoek^1,3^; Dutch Meningioma Consortium

*equal contribution

*Dutch Meningioma Consortium: N.R. Biermasz^7^, F.W. Boele^8^, L. Dirven^1^, M. Klein^9^, W.A. Moojen^4,10,11^, J.C. Reijneveld^12,13^, M.J.T. Verstegen^4^*

*^1^ Department of Neurology, Leiden University Medical Center, Leiden, the Netherlands*

*^2^Department of Ophthalmology, Leiden University Medical Center, Leiden, the Netherlands*

*^3^Department of Neurology, Haaglanden Medical Center, The Hague, the Netherlands*

*^4^ Department of Neurosurgery, Leiden University Medical Center, Leiden, the Netherlands*

*^5^ Department of Neurosurgery, Amsterdam University Medical Center-VU University Amsterdam, the Netherlands*

*^6^ Department of Psychiatry, University Medical Center Utrecht, Utrecht, The Netherlands*

*^7^ Department of Internal Medicine, Leiden University Medical Center, Leiden, the Netherlands*

*^8^ Department of Medical Psychology, University of Leeds, Leeds, United Kingdom*

*^9^ Department of Medical Psychology, Amsterdam University Medical Center-VU University Amsterdam, the Netherlands*

*^10^ Department of Neurosurgery, Haaglanden Medical Center, Leiden, the Netherlands*

*^11^ Department of Neurosurgery, HAGA Hospital, The Hague, the Netherlands*

*^12^Department of Neurology, SEIN, Heemstede, the Netherlands*

*^13^ Department of Neurology, Amsterdam University Medical Center-VU University Amsterdam, the Netherlands*

Corresponding Author: L. Laribi, BSc, Department of Neurology, Leiden University Medical Center, Postbus 9600, 2300 RC Leiden, the Netherlands, telephone +31 (0)71 526 21 97, email [l.laribi@umail.leidenuniv.nl](mailto:l.laribi@umail.leidenuniv.nl), ORCID - 0009-0009-1103-2458

Supplementary Material

| **Supplementary Table 1. STROBE checklist of items that should be included in reports of observational studies [45][46]** | | |
| --- | --- | --- |
| **Title and abstract** | (*a*) Indicate the study’s design with a commonly used term in the title or the abstract | **✓** |
|  | (*b*) Provide in the abstract an informative and balanced summary of what was done and what was found | **✓** |
| **Introduction** | | |
| Background/rationale | Explain the scientific background and rationale for the investigation being reported | **✓** |
| Objectives | State specific objectives, including any prespecified hypotheses | **✓** |
| **Methods** | | |
| Study design | Present key elements of study design early in the paper | **✓** |
| Setting | Describe the setting, locations, and relevant dates, including periods of recruitment, exposure, follow-up, and data collection | **✓** |
| Participants | (*a*) *Cohort study*—Give the eligibility criteria, and the sources and methods of selection of participants. Describe methods of follow-up  *Case-control study*—Give the eligibility criteria, and the sources and methods of case ascertainment and control selection. Give the rationale for the choice of cases and controls  *Cross-sectional study*—Give the eligibility criteria, and the sources and methods of selection of participants | **✓** |
|  | (*b*) *Cohort study*—For matched studies, give matching criteria and number of exposed and unexposed  *Case-control study*—For matched studies, give matching criteria and the number of controls per case | **NA** |
| Variables | Clearly define all outcomes, exposures, predictors, potential confounders, and effect modifiers. Give diagnostic criteria, if applicable | **✓** |
| Data sources/ measurement | For each variable of interest, give sources of data and details of methods of assessment (measurement). Describe comparability of assessment methods if there is more than one group | **NA** |
| Bias | Describe any efforts to address potential sources of bias | **✓** |
| Study size | Explain how the study size was arrived at | **X** |
| Quantitative variables | Explain how quantitative variables were handled in the analyses. If applicable, describe which groupings were chosen and why | **NA** |
| Statistical methods | (*a*) Describe all statistical methods, including those used to control for confounding | **✓** |
|  | (*b*) Describe any methods used to examine subgroups and interactions | **✓** |
|  | (*c*) Explain how missing data were addressed | **✓** |
|  | (*d*) *Cohort study*—If applicable, explain how loss to follow-up was addressed  *Case-control study*—If applicable, explain how matching of cases and controls was addressed  *Cross-sectional study*—If applicable, describe analytical methods taking account of sampling strategy | **NA** |
|  | (*e*) Describe any sensitivity analyses | **✓** |
| Participants | (a) Report numbers of individuals at each stage of study—eg numbers potentially eligible, examined for eligibility, confirmed eligible, included in the study, completing follow-up, and analysed | **✓** |
|  | (b) Give reasons for non-participation at each stage | **NA** |
|  | (c) Consider use of a flow diagram | **NA** |
| Descriptive data | (a) Give characteristics of study participants (eg demographic, clinical, social) and information on exposures and potential confounders | **✓** |
|  | (b) Indicate number of participants with missing data for each variable of interest | **✓** |
|  | (c) *Cohort study*—Summarise follow-up time (eg, average and total amount) | **NA** |
| Outcome data | *Cohort study*—Report numbers of outcome events or summary measures over time | **✓** |
|  | *Case-control study—*Report numbers in each exposure category, or summary measures of exposure | **✓** |
|  | *Cross-sectional study—*Report numbers of outcome events or summary measures | **✓** |
| Main results | (*a*) Give unadjusted estimates and, if applicable, confounder-adjusted estimates and their precision (eg, 95% confidence interval). Make clear which confounders were adjusted for and why they were included | **✓** |
|  | (*b*) Report category boundaries when continuous variables were categorized | **NA** |
|  | (*c*) If relevant, consider translating estimates of relative risk into absolute risk for a meaningful time period | **NA** |
| Other analyses | Report other analyses done—eg analyses of subgroups and interactions, and sensitivity analyses | **✓** |
| Key results | Summarise key results with reference to study objectives | **✓** |
| Limitations | Discuss limitations of the study, taking into account sources of potential bias or imprecision. Discuss both direction and magnitude of any potential bias | **✓** |
| Interpretation | Give a cautious overall interpretation of results considering objectives, limitations, multiplicity of analyses, results from similar studies, and other relevant evidence | **✓** |
| Generalisability | Discuss the generalisability (external validity) of the study results | **✓** |
| **Other information** | | |
| Funding | Give the source of funding and the role of the funders for the present study and, if applicable, for the original study on which the present article is based | **✓** |

**Supplementary Table 2. Medications prescribed to meningioma patients in our study with corresponding neuropsychiatric and neurocognitive adverse effects**

|  |  | **Adverse effects** | | |
| --- | --- | --- | --- | --- |
| **Medication** | **No. of patients (n=187** | **Depressive^1^** | **Anxiety^2^** | **Cognitive^3^** |
| Acenocoumarol | 3 | - | - | - |
| Acetic acid | 1 | - | - | - |
| Acetylcysteine | 2 | - | Reported | - |
| Acetylsalicyl acid | 9 | - | Reported | Reported |
| Alendronic acid* | 8 | - | - | - |
| Alfuzosin | 2 | - | - | - |
| Allopurinol | 1 | <0,01% | - | - |
| Almotriptan | 1 | - | - | - |
| Aluminiumhydroxychloride | 1 | - | - | - |
| Amantadine | 1 | **>1%** | **>1%** | **>1%** |
| Amiodarone | 1 | **-** | **-** | Reported |
| Amitryptiline** | 3 | 0,01-0,1% | **>1%** | **>1%** |
| Amlodipine | 7 | 0,1-1% | - | 0,01-0,1% |
| Amoxicilline/clavulanic acid | 1 | - | - | - |
| Atenolol | 1 | 0,1-1% | **-** | 0,1-1% |
| Atorvastatin | 8 | Reported | - | - |
| Azathioprine | 2 | - | - | - |
| Beclomethasone | 2 | Reported | Reported | - |
| Betahistine | 1 | - | - | - |
| Bisoprolol | 1 | 0,1-1% | - | - |
| Brinzolamide/brimonidine | 1 | - | - | - |
| Brinzolamide/timolol | 1 | Reported | - | - |
| Budesonide | 3 | 0,1-1% | 0,1-1% | - |
| Bumetanide | 3 | - | - | - |
| Calcium carbonate | 9 | - | - | - |
| Calcium carbonate/colecalciferol | 10 | - | - | - |
| Candesartan | 6 | - | - | - |
| Carbamazepine**^/4^ | 11 | 0,1-1% | - | <0,01% |
| Carbasalate calcium | 7 | - | - | - |
| Carbomer | 6 | - | - | - |
| Carboxymethylcellulose | 1 | - | - | - |
| Cetirizine | 1 | 0,01-0,1% | - | 0,01-0,1% |
| Chloortalidon | 1 | - | - | - |
| Cholecalciferol or colecalciferol | 17 | - | - | - |
| Citalopram**^/^*** | 1 | - | **>1%** | **>1%** |
| Clindamycin | 1 | - | - | - |
| Clobazam** | 3 | **>1%** | 0,1-1% | **>1%** |
| Clomipramine**^/^*** | 1 | **>1%** | **>1%** | **>1%** |
| Clonazepam | 2 | **>1%** | **>1%** | **>1%** |
| Clopidogrel | 3 | - | - | <0,01% |
| Codeine | 1 | Reported | Reported | Reported |
| Dabigatran | 1 | - | - | - |
| Darifenacin | 1 | Reported | - | 0.1-1% |
| Denosumab | 1 | - | - | - |
| Dexamethasone | 1 | **>1%** | **>1%** | - |
| Dextran/hypromellose | 3 | - | - | - |
| Diazepam*** | 3 | **>1%^6^** | - | **>1%** |
| Diclofenac* | 6 | <0,01% | <0,01% | <0,01% |
| Diclofenac/misoprostol | 1 | 0.1%-1% | 0.1%-1% | Reported |
| Digoxin | 1 | 0.1%-1% | - | <0.01% |
| Dipyridamol | 4 | - | - | - |
| Dorzolamide/​timolol | 1 | - | - | - |
| Doxazosin | 1 | 0,1-1% | 0,1-1% | - |
| Emedastin | 1 | - | - | - |
| Enalapril | 4 | **>1%** | 0,1-1% | 0,1-1% |
| Escitalopram**^/^*** | 1 | **-** | **>1%** | - |
| Esomeprazole | 8 | 0,01-0,1% | - | 0,01-0,1% |
| Estradiol | 1 | **>1%** | 0,1-1% | - |
| Estriol | 1 | **-** | - | - |
| Ethinylestradiol/levonorgestrel | 1 | **>1%** | **>1%^7^** | - |
| Ezetimibe | 1 | - | - | - |
| Feneticilline | 1 | - | - | - |
| Fexofenadine | 2 | - | - | - |
| Finasteride | 1 | 0,1-1% | - | - |
| Flecainide | 1 | 0,01-0,1% | 0,1-1% | 0,1-1% |
| Flunitrazepam | 1 | Reported | - | Reported |
| Fluoxetine**^/^*** | 1 | 0,1-1% | **>1%** | **>1%** |
| Fluticasone | 10 | <0,01% | <0,01% | - |
| Folic acid | 1 | <0,01% | - | - |
| Formoterol | 1 | - | 0,1-1% | - |
| Formoterol/budesonide | 3 | <0.01% | 0.1-1% | - |
| Furosemide | 2 | - | - | 0.01-0.1% |
| Fusidic acid | 1 | - | - | - |
| Gabapentin | 3 | **>1%** | **>1%** | **>1%** |
| Glimepiride | 2 | - | - | - |
| Hyaluronic acid/carbomer | 1 | - | - | - |
| Hydrochlorothiazide | 15 | 0,01-0,1% | - | - |
| Hydrocortisone | 3 | **>1%** | Reported | - |
| Hydroquinine | 1 | - | - | - |
| Hydroxocobalamine | 4 | - | - | - |
| Hydroxychloroquine | 4 | - | 0,01-0,1% | - |
| Hypromellose | 1 | - | - | - |
| Indifferente (vette) creme | 1 | - | - | - |
| Insulin agents | 5 | - | - | - |
| Ipratropium | 1 | - | - | - |
| Irbesartan | 5 | - | - | - |
| Isosorbide mononitrate | 3 | - | - | - |
| Ketotifen | 1 | - | - | - |
| Lactulose | 2 | - | - | - |
| Lamotrigine** | 8 | - | - | <0,01% |
| Lansoprazol | 1 | 0.1-1% | - | 0.01-0.1% |
| Latanoprost | 2 | - | - | - |
| Lercanidipine | 3 | - | - | - |
| Levetiracetam | 14 | **>1%** | **>1%** | 0,1-1% |
| Levocetirizine | 2 | Reported | Reported | - |
| Levonorgestrel | 1 | **>1%** | **>1%^7^** | - |
| Levothyroxine | 16 | - | - | - |
| Lidocaine/propylparahydroxybenzoaat | 1 | - | - | - |
| Linagliptine | 1 | - | - | - |
| Lisinopril | 6 | 0,1-1% | - | 0,01-0,1% |
| Loratadine | 1 | - | **>1%^7^** | - |
| Losartan | 9 | Reported | - | - |
| Macrogol | 4 | - | - | - |
| Magnesium hydroxide | 2 | - | - | - |
| Mesalazine | 2 | Reported | - | - |
| Metformin | 11 | - | - | - |
| Metoprolol | 19 | 0,1-1% | 0,01-0,1% | 0,1-1% |
| Metronidazole | 2 | - | - | - |
| Mirabegron | 1 | - | - | Reported |
| Mirtazapine** | 2 | Reported | **>1%** | **>1%** |
| Mometasone | 2 | - | - | - |
| Montelukast | 1 | 0,1-1% | 0,1-1% | 0,01-0,1% |
| Morphine | 1 | 0,1-1% | - | **>1%** |
| Naproxen | 1 | 0,01-0,1% | - | 0,01-0,1% |
| Nifedipine | 4 | <0,01% | 0,1-1% | - |
| Nitrazepam | 1 | Reported | Reported | Reported |
| Nitrofurantoin | 1 | Reported | - | - |
| Nitroglycerine | 3 | - | - | - |
| Omeprazole* | 17 | 0,01-0,1% | 0,01-0,1% | - |
| Ondansetron | 1 | - | - | - |
| Oxazepam*** | 3 | **>1%^6^** | - | **>1%** |
| Oxcarbazepine | 2 | **>1%** | - | **>1%** |
| Oxytocine | 1 | **-** | **-** | **-** |
| Pantoprazole | 16 | 0,01-0,1% | - | <0,01% |
| Paracetamol^5^ | 11 | 0,1-1% | - | 0,1-1% |
| Paracetamol/codeïne | 1 | 0,01-0,1% | Reported | 0,01-0,1% |
| Paracetamol/​propyfenazon/​coffeïne | 1 | 0.01-0.1% | - | 0.01-0,1% |
| Paracetamol/tramadol | 1 | **>1%** | **>1%** | **>1%** |
| Paroxetine**^/^*** | 9 | Reported | **>1%^7^** | **>1%** |
| Perindopril | 7 | 0,1-1% | - | <0,01% |
| Phenprocoumon | 5 | - | - | - |
| Phenytoin | 1 | - | **>1%^7^** | **>1%** |
| Pilocarpine | 1 | - | **-** | **-** |
| Povidon | 3 | - | - | - |
| Pramipexole | 2 | Reported | Reported | **>1%** |
| Prasterone^8^ | 1 | - | - | **-** |
| Pravastatin | 4 | Reported | - | Reported |
| Prednisolone | 3 | **>1%** | **>1%** | - |
| Pregabalin*** | 1 | 0,1-1% | 0,1-1% | **>1%** |
| Promethazine | 1 | Reported | - | **-** |
| Propranolol*** | 3 | 0,01-0,1% | - | 0,01-0,1% |
| Psyllium husk | 4 | - | - | - |
| Ranitidine | 3 | <0,01% | - | <0,01% |
| Risedronate | 1 | - | - | - |
| Rivaroxaban | 2 | - | - | - |
| Rizatriptan | 1 | - | - | 0.1-1% |
| Rosuvastatin | 2 | Reported | **-** | <0.01% |
| Salbutamol | 5 | - | - | - |
| Salmeterol | 1 | - | - | - |
| Salmeterol/fluticasone | 1 | - | 0,1-1% | - |
| Simvastatin | 27 | Reported | - | <0,01% |
| Somatropine | 1 | 0,01-0,1% | - | - |
| Sotalol | 8 | **>1%** | **>1%** | - |
| Spironolactone | 2 | - | - | 0,1-1% |
| Sucralfate | 1 | - | - | - |
| Sumatriptan | 1 | - | - | - |
| Tacrolimus | 1 | **>1%** | **>1%** | **>1%** |
| Tafluprost | 1 | **-** | **-** | **-** |
| Tamsulosin | 3 | - | - | - |
| Telmisartan | 2 | 0.1-1% | 0.01-0.1% | - |
| Temazepam | 5 | **>1%^6^** | - | **>1%** |
| Testosterone | 2 | **>1%^6^** | Reported | - |
| Tibolon | 2 | Reported | - | - |
| Timolol/bimatoprost | 2 | 0,1-1% | - | 0,01-0,1% |
| Tiotropium/olodaterol | 1 | - | - | - |
| Topiramate | 1 | **>1%** | **>1%** | **>1%** |
| Tramadol | 5 | 0,01-0,1% | 0,01-0,1% | 0,01-0,1% |
| Trazodone**^/^*** | 1 | Reported | **>1%^7^** | **>1%** |
| Triamcinolone | 2 | - | - | - |
| Ureum | 1 | - | - | - |
| Valproic acid** | 9 | Reported | - | **>1%** |
| Vaseline/paraffine | 1 | - | - | **-** |
| Venlafaxine**^/^*** | 1 | 0,1-1% | - | **>1%** |
| Zinc oxide | 1 | - | - | - |
| Zopiclone | 1 | 0.01-0.1% | 0.01-0.1% | 0.01-0.1% |

^1^The following adverse effects were considered depressive: depression, flat affect, lethargy, apathy, suicidal thoughts, mood disorder and mood swings.

^2^The following adverse effects were considered anxiety: anxiety, nervousness, agitation and panic (attacks).

^3^The following adverse effects were considered cognitive: cognitive impairment, concentration disorder, confusion, amnesia, attention disorder, reduced memory, bradyphrenia, aphasia, reduced alertness and disorientation.

^4^Carbamazepine was considered as a medication with >1% risk of cognitive adverse effects, despite the percentages on Farmacotherapeutisch Kompas, based on literature;(1, 2).

^5^We did not include paracetamol in our analyses, as we were not confident this was reported adequately in the medical record.

^6^Closely related adverse effect of depression and considered depressive, only indicated if >1%.

^7^Closely related adverse effect of anxiety and considered as an anxiety adverse effect, only indicated if >1%.

^8^Prasterone was considered as a medication with no risk of cognitive adverse effects based on the Belgian Federal Agency for Medicines and Health Products [47] and due to its absence on Farmacotherapeutisch Kompas.

*Mentions of a medical group instead of an exact medication was included as the most commonly prescribed drug of said group.

**These medications have a mood stabilizing indication according to Farmacotherapeutisch Kompas; ***These medications have an anxiolytic indication according to Farmacotherapeutisch Kompas.

| **Supplementary Table 3: Summary of non-response analysis** | | | | | |
| --- | --- | --- | --- | --- | --- |
|  | | Response (N = 187) | Non-Response (N = 450) | Total (N = 637) | p-value |
| Gender  [n, %] | Male (ref.) | 40 (21.4%) | 105 (23.3%) | 145 (22.8%) | 0.566 |
|  | Female | 147 (78.6%) | 342 (76%) | 489 (76.8%) |  |
|  | Unknown | 0 (0.00%) | 3 (0.7%) | 3 (0.5%) |  |
| Age at study date (years)  [mean, SD] | | 63.15 (11.08) | 66.96 (13.52) |  | < 0.001* |
| WHO grade  [n, %] | Grade I (ref.) | 146 (78%) | 291 (65%) | 437 (69%) | 0.524 |
|  | Grade II - III | 12 (6%) | 30 (7%) | 42 (7%) |  |
|  | Unknown | 29 (16%) | 129 (29%) | 158 (25%) |  |
| Radiotherapy  [n, %] | No (ref.) | 153 (81.8%) | 324 (72%) | 477 (74.9%) | 0.035* |
|  | Yes | 34 (18.2%) | 114 (25.3%) | 148 (23.2%) |  |
|  | Unknown | 0 (0.00%) | 12 (2.7%) | 12 (1.9%) |  |
| Surgery  [n, %] | No (ref.) | 23 (12.3%) | 91 (20.2%) | 114 (17.9%) | 0.012* |
|  | Yes | 164 (87.7%) | 348 (77.3%) | 512 (80.4%) |  |
|  | Unknown | 0 (0.00%) | 11 (2.4%) | 11 (1.7%) |  |
| *ref.* = reference category  * p-value < 0.05 | | | | | |

| **Supplementary Table 4: Odds ratios of ASM use and the selected confounders per outcome in the univariable analysis** | | | | | | |
| --- | --- | --- | --- | --- | --- | --- |
|  | Depression^1^ | | | | | |
|  | uOR | 95% CI | P-value | aOR | 95% CI | P-value |
| ASM use | 1.021 | 0.405 - 2.572 | 0.965 | 0.812 | 0.260 – 2.538 | 0.720 |
| Age | 0.996 | 0.962 – 1.032 | 0.841 | 0.955 | 0.912 – 1.000 | 0.049 |
| WHO grade | 1.052 | 0.216 – 5.120 | 0.950 | 1.167 | 0.223 – 6.114 | 0.855 |
| Treatment (Radiotherapy and/or Surgery) | 0.372 | 0.105 - 1.324 | 0.127 | N/A | N/A | N/A |
| Seizures in the last three months | 1.114 | 0.948 - 1.308 | 0.189 | 0.721 | 0.220 – 2.362 | 0.589 |
| Use of medication with neuropsychiatric and/or neurocognitive related adverse effects^a^ | 2.435 | 1.086 – 5.457 | 0.031* | 2.889 | 1.119 – 7.456 | 0.028* |
|  | Anxiety^1^ | | | | | |
|  | uOR | 95% CI | P-value | aOR | 95% CI | P-value |
| ASM use | 0.647 | 0.264 - 1.588 | 0.342 | 0.633 | 0.220 – 1.821 | 0.397 |
| Age | 0.970 | 0.940 - 1.001 | 0.059 | 0.951 | 0.914 – 0.990 | 0.015* |
| WHO grade | 1.177 | 0.301 – 4.606 | 0.815 | 1.332 | 0.312 – 5.686 | 0.699 |
| Treatment (Radiotherapy and/or Surgery) | 0.970 | 0.254 – 3.700 | 0.965 | N/A | N/A | N/A |
| Seizures in the last three months | 0.616 | 0.168 - 2.260 | 0.465 | 0.667 | 0.183 – 2.430 | 0.540 |
| Use of medication with neuropsychiatric and/or neurocognitive related adverse effects^a^ | 2.850 | 1.371 – 5.923 | 0.005* | 2.860 | 1.199 – 6.822 | 0.018* |
|  | Cognitive Impairment^2^ | | | | | |
|  | uOR | 95% CI | P-value | aOR | 95% CI | P-value |
| ASM use | 0.885 | 0.397 - 1.973 | 0.765 | 1.420 | 0.506 – 3.984 | 0.505 |
| Age | 1.087 | 1.047 – 1.129 | < 0.001* | 1.092 | 1.039 – 1.147 | < 0.001* |
| WHO grade | 0.685 | 0.143 – 3.282 | 0.636 | 0.547 | 0.096 – 3.108 | 0.496 |
| Treatment (Radiotherapy and/or Surgery) | 0.273 | 0.087 – 0.856 | 0.026* | N/A | N/A | N/A |
| Seizures in the last three months | 0.745 | 0.348 - 1.593 | 0.448 | 0.670 | 0.263 – 1.709 | 0.402 |
| Use of medication with neuropsychiatric and/or neurocognitive related adverse effects^a^ | 1.834 | 0.900 – 3.734 | 0.095 | 2.179 | 0.901 – 5.272 | 0.084 |
| The unadjusted odds ratios (uOR) and adjusted odds ratios (aOR) with corresponding 95% confidence intervals (CI) and p-values for ASM users versus non-users with regards to each outcome variable.  N/A = Not Applicable  ^1^ ≥ 8 on the HADS-D ^2^ ≤ -1.5 in at least 2 of the neurocognitive domains  * p-value < 0.05 ^a^ Excluding ASMs | | | | | | |

| **Supplementary Table 5: Odds ratios of ASM use and the selected confounders per neurocognitive domain in the multivariable analysis** | | | | | | |
| --- | --- | --- | --- | --- | --- | --- |
|  | Executive Functioning and Psychomotor Functioning (≤ -1.5 on CST and CWFT) | | | | | |
|  | uOR | 95% CI | P-value | aOR | 95% CI | P-value |
| ASM use | 1.296 | 0.532 - 3.162 | 0.568 | 2.408 | 0.765 – 7.577 | 0.133 |
| Age | 1.087 | 1.041 – 1.135 | < 0.001* | 1.062 | 1.005 – 1.122 | 0.033* |
| WHO grade | 0.573 | 0.070 – 4.681 | 0.603 | 0.505 | 0.052 – 4.921 | 0.556 |
| Treatment (Radiotherapy and/or Surgery) | 0.196 | 0.061 – 0.631 | 0.006* | N/A | N/A | N/A |
| Seizures in the last three months | 0.904 | 0.598 – 1.367 | 0.634 | 0.834 | 0.457 – 1.522 | 0.554 |
| Use of medication with neuropsychiatric and/or neurocognitive related adverse effects^a^ | 3.494 | 1.566 – 7.795 | 0.002* | 4.081 | 1.510 – 11.026 | 0.006* |
|  | Verbal Memory (≤ -1.5 on CST and AVLT) | | | | | |
|  | uOR | 95% CI | P-value | aOR | 95% CI | P-value |
| ASM use | 2.286 | 0.885 - 5.903 | 0.088 | 5.607 | 1.710– 18.384 | 0.004* |
| Age | 1.041 | 0.997 – 1.087 | 0.067 | 0.994 | 0.935 – 1.057 | 0.849 |
| WHO grade | 0.930 | 0.111 – 7.786 | 0.947 | 0.667 | 0.069 – 6.487 | 0.727 |
| Treatment (Radiotherapy and/or Surgery) | 0.173 | 0.051 – 0.589 | 0.005* | N/A | N/A | N/A |
| Seizures in the last three months | 0.000 | 0.000 | 0.996 | 0.000 | 0.000 | 0.997 |
| Use of medication with neuropsychiatric and/or neurocognitive related adverse effects^a^ | 1.411 | 0.538 – 3.701 | 0.484 | 2.521 | 0.759 – 8.372 | 0.131 |
|  | Working Memory (≤ -1.5 on MST) | | | | | |
|  | uOR | 95% CI | P-value | aOR | 95% CI | P-value |
| ASM use | 1.134 | 0.448 - 2.873 | 0.790 | 1.546 | 0.460 – 5.196 | 0.481 |
| Age | 1.044 | 1.004 – 1.085 | 0.029* | 1.034 | 0.980 – 1.090 | 0.221 |
| WHO grade | 1.506 | 0.304 – 7.461 | 0.616 | 1.780 | 0.308 – 10.296 | 0.520 |
| Treatment (Radiotherapy and/or Surgery) | 0.398 | 0.114 – 1.388 | 0.148 | N/A | N/A | N/A |
| Seizures in the last three months | 0.000 | 0.000 | 0.996 | 0.000 | 0.000 | 0.997 |
| Use of medication with neuropsychiatric and/or neurocognitive related adverse effects^a^ | 3.140 | 1.394 – 7.070 | 0.006* | 4.529 | 1.603 – 12.796 | 0.004* |
|  | Attention (≤ -1.5 on SCWT) | | | | | |
|  | uOR | 95% CI | P-value | aOR | 95% CI | P-value |
| ASM use | 1.629 | 0.741 - 3.577 | 0.224 | 2.527 | 0.885 – 7.219 | 0.083 |
| Age | 1.106 | 1.060 – 1.155 | < 0.001* | 1.115 | 1.055 – 1.178 | < 0.001* |
| WHO grade | 2.054 | 0.577 – 7.307 | 0.267 | 1.837 | 0.397 – 8.837 | 0.428 |
| Treatment (Radiotherapy and/or Surgery) | 0.442 | 0.136 – 1.431 | 0.173 | N/A | N/A | N/A |
| Seizures in the last three months | 1.004 | 0.852 – 1.182 | 0.966 | 1.076 | 0.760 – 1.523 | 0.680 |
| Use of medication with neuropsychiatric and/or neurocognitive related adverse effects^a^ | 1.989 | 0.943 – 4.196 | 0.071 | 2.090 | 0.802 – 5.445 | 0.131 |
|  | Information Processing (≤ -1.5 on DSST) | | | | | |
|  | uOR | 95% CI | P-value | aOR | 95% CI | P-value |
| ASM use | 1.068 | 0.486 - 2.345 | 0.870 | 1.547 | 0.598 – 4.003 | 0.368 |
| Age | 1.050 | 1.016 – 1.068 | 0.004* | 1.038 | 0.996 – 1.082 | 0.078 |
| WHO grade | 1.121 | 0.287 – 4.382 | 0.869 | 1.039 | 0.246 – 4.391 | 0.959 |
| Treatment (Radiotherapy and/or Surgery) | 0.389 | 0.124 – 1.221 | 0.106 | N/A | N/A | N/A |
| Seizures in the last three months | 0.614 | 0.179 – 2.101 | 0.437 | 0.523 | 0.121 – 2.267 | 0.386 |
| Use of medication with neuropsychiatric and/or neurocognitive related adverse effects^a^ | 1.642 | 0.800 – 3.371 | 0.176 | 1.840 | 0.784 – 4.317 | 0.161 |
|  | Processing Speed (≤ -1.5 on CST and DSST) | | | | | |
|  | uOR | 95% CI | P-value | aOR | 95% CI | P-value |
| ASM use | 0.958 | 0.400 - 2.294 | 0.924 | 1.746 | 0.604 – 5.047 | 0.303 |
| Age | 1.063 | 1.023 – 1.103 | 0.002* | 1.034 | 0.986 – 1.084 | 0.167 |
| WHO grade | 0.394 | 0.049 – 3.183 | 0.382 | 0.369 | 0.041 – 3.319 | 0.374 |
| Treatment (Radiotherapy and/or Surgery) | 0.380 | 0.117 – 1.236 | 0.108 | N/A | N/A | N/A |
| Seizures in the last three months | 0.000 | 0.000 | 0.996 | 0.000 | 0.000 | 0.997 |
| Use of medication with neuropsychiatric and/or neurocognitive related adverse effects^a^ | 2.721 | 1.278 – 5.797 | 0.009* | 3.609 | 1.472 – 8.846 | 0.005* |
| The unadjusted odds ratios (uOR) and adjusted odds ratios (aOR) with corresponding 95% confidence intervals (CI) and p-values for ASM users versus non-users with regards to each outcome variable per neurocognitive domain.  N/A = Not Applicable  * p-value < 0.05 ^a^ Excluding ASMs | | | | | | |
